# RAB39B as a Chemosensitivity-Related Biomarker for Diffuse Large B-Cell Lymphoma

**DOI:** 10.3389/fphar.2022.931501

**Published:** 2022-07-15

**Authors:** Cong Xu, Ting Liang, Jing Liu, Yunfeng Fu

**Affiliations:** ^1^ Department of Hematology, The Third Xiangya Hospital of Central South University, Changsha, China; ^2^ Department of Hematology, The Seventh Affiliated Hospital of Sun Yat-Sen University, Shenzhen, China; ^3^ Department of Blood Transfusion, The Seventh Affiliated Hospital of Sun Yat-Sen University, Shenzhen, China; ^4^ Department of Blood Transfusion, The Third Xiangya Hospital of Central South University, Changsha, China

**Keywords:** RAB39B, diffuse large B-cell lymphoma, immune infiltration, m6A modification, drug sensitivity

## Abstract

**Background:** Diffuse large B-cell lymphoma (DLBCL) is the most common aggressive lymphoma with an increased tendency to relapse or refractoriness. RAB39B, a member of the Ras-oncogene superfamily, is associated with a variety of tumors. Nevertheless, the role of RAB39B in DLBCL is still unknown. This study aimed to identify the role of RAB39B in DLBCL using integrated bioinformatics analysis.

**Methods:** RAB39B expression data were examined using TIMER, UCSC, and GEO databases. The LinkedOmics database was used to study the genes and signaling pathways related to RAB39B expression. A Protein–protein interaction network was performed in STRING. TIMER was used to analyze the correlation between RAB39B and infiltrating immune cells. The correlation between RAB39B and m6A-related genes in DLBCL was analyzed using TCGA data. The RAB39B ceRNA network was constructed based on starBase and miRNet2.0 databases. Drug sensitivity information was obtained from the GSCA database.

**Results:** RAB39B was highly expressed in multiple tumors including DLBCL. The protein–protein interaction network showed enrichment of autophagy and RAS family proteins. Functional enrichment analysis of RAB39B co-expression genes revealed that RAB39B was closely related to DNA replication, protein synthesis, cytokine–cytokine receptor interaction, JAK-STAT signaling pathway, NF-kappa B signaling pathway, and autophagy. Immune infiltrate analysis showed that the amount of RAB39B was negatively correlated with iDC, Tem, and CD8 T-cell infiltration. CD4^+^ T cell and DC were negatively correlated with CNV of RAB39B. DLBCL cohort analysis found that RAB39B expression was related to 14 m6A modifier genes, including YTHDC1, YTHDC2, YTHDF1, YTHDF2, YTHDF3, RBMX, ZC3H13, METTL14, METTL3, RBM15, RBM15B, VIRMA, FTO, and ALKBH5. We constructed 14 possible ceRNA networks of RAB39B in DLBCL. The RAB39B expression was associated with decreased sensitivity of chemotherapy drugs such as dexamethasone, doxorubicin, etoposide, vincristine, and cytarabine and poor overall survival in DLBCL. *In vitro* experiments showed that RAB39B was associated with proliferation, apoptosis, and drug sensitivity of DLBCL cells.

**Conclusion:** RAB39B is abnormally elevated and related to drug resistance and poor OS in DLBCL, which may be due to its involvement in immune infiltration, m6A modification, and regulation by multiple non-coding RNAs. RAB39B may be used as an effective biomarker for the diagnosis and treatment of DLBCL.

## Introduction

Diffuse large B-cell lymphoma (DLBCL) is the most common non-Hodgkin’s lymphoma, accounting for around 75% of aggressive lymphomas in adults (Teras et al., 2016). It is histologically characterized by diffuse growth, nodal architectural destruction, and extranodal infiltration with large B lymphoid cells. Although typically R-CHOP (rituximab plus cyclophosphamide, doxorubicin, vincristine, and prednisone)-based chemotherapy cures a majority of patients with DLBCL, ∼40% of the patients either suffer a relapse or have primary refractory disease (Coiffier et al., 2002). Although remarkable progress has been made in understanding the pathogenesis, the precise mechanism of DLBCL remains unknown. The recently revised World Health Organization (WHO) classification of DLBCL includes the germinal center B cell–like (GCB) and non-GCB [which includes activated B cell–like (ABC) and unclassified by gene expression profiling (GEP)] cell types as new entities based on IHC staining (Swerdlow et al., 2016). Non-GCB DLBCL is associated with higher CNS relapse risk and poor prognosis (Offner et al., 2015; Klanova et al., 2019). Gaining an in-depth understanding of the mechanisms that drive DLBCL pathogenesis and response to therapy has important practical and theoretical value.

The pathogenesis of DLBCL represents a multi-factorial process. Modern genome-wide molecular analysis has uncovered significant implications of gene mutation, epigenetic remodeling, differentiation block, immune surveillance escape, immune infiltration, and the constitutive activation of several signal transduction pathways in the initiation and maintenance of the tumor clone in DLBCL (Pasqualucci and Dalla-Favera, 2018; Cheng et al., 2020; Lee et al., 2020). Similar to most cancer, several mechanisms contribute to oncogenic dysregulation in DLBCL, including gene copy number changes and somatically acquired non-silent point mutations. In addition, the genome of DLBCL is altered by chromosomal translocations and aberrant somatic hypermutation, both of which are intimately connected to the physiologic IG DNA remodeling processes operating in B lymphocytes (Pasqualucci and Dalla-Favera, 2018). Among them, the canonical cancer-related RAS/RAF/MEK/ERK pathway is considered to be associated with DLBCL cell proliferation, migration, invasion, drug sensitivity, and prognosis (Jiang et al., 2018; Yang et al., 2020; Sun et al., 2021; Wang and Sun, 2021).

RAB39B, a member of the Ras-oncogene superfamily, consists of two exons spanning 3764 bp of human genomic DNA and locates in human chromosome Xq28 (Cheng et al., 2002). RAB39B is expressed in multiple human tissues. Previous studies have mainly focused on the role of RAB39B abnormalities in X-linked neurodevelopmental defects including macrocephaly, intellectual disability, autism spectrum disorder, and Parkinson’s disease (Tang, 2021). In cancer research, RAB39B is reported to be upregulated in germ cell neoplastic and gastric stromal tumors (Biermann et al., 2007; Kou et al., 2015). Studies have also shown that RAB39B is correlated with immune-infiltrating cells and poor overall survival in pancreatic adenocarcinoma (He et al., 2020). RAB39B has been considered to be involved in the regulation of autophagy and the PI3K/Akt/mTOR signaling pathway (Giannandrea et al., 2010; Tang, 2021). However, the underlying molecular mechanisms involved remain largely unknown and require detailed characterization. To date, there are no data on the expression and biological function of RAB39B in DLBCL.

In this study, we analyzed the differences in the expression of RAB39B in DLBCL from various public databases. Protein–protein interaction (PPI), co-expressed genes, immune infiltration, N6-methyladenosine (m6A) RNA methylation, and ceRNA networks were used to evaluate the potential mechanism of RAB39B in DLBCL. We also evaluated the impact of RAB39B expression on cell proliferation, apoptosis, drug sensitivity, and prognosis of DLBCL. This study provides a theoretical basis for the role of RAB39B in DLBCL.

## Materials and Methods

### Tumor Immune Estimation Resource Analysis

Tumor Immune Estimation Resource (TIMER, https://cistrome.shinyapps.io/timer/) is a reliable and convenient database including comprehensive immune infiltrates and gene expression resources across diverse cancer types and TCGA gene expression profiles (Li et al., 2016; Li T. et al., 2017). In this study, we evaluated the mRNA level of RAB39B between tumor and adjacent normal tissues in pan-cancer through the DiffExp module of the TIMER database. The somatic copy number alteration (SCNA) module was used to link the genetic copy number variations (CNVs) of RAB39B with the relative abundance of immune-infiltrating cells.

### UCSC Xena Data

DLBCL RNA-seq data from TCGA database and RNA-seq data of normal samples from the GTEx database were downloaded from UCSC Xena (https://xenabrowser.net/datapages/) (Goldman et al., 2020). We obtained 47 tumor samples and 444 normal samples for analysis.

### Gene Expression Omnibus Data

We downloaded GSE9327 (*n* = 200) RNA-seq data from the Gene Expression Omnibus (GEO, www.ncbi.nlm.nih.gov/geo) database, which included 8 reactive lymph nodes and 36 DLBCL samples.

### The Cancer Genome Atlas Data

The Cancer Genome Atlas (TCGA, https://portal.gdc.cancer.gov/) characterizes more than 20,000 samples of 33 cancer types (Tomczak et al., 2015). DLBCL RNA-seq data were downloaded from the Genomic Data Commons (GDC, https://portal.gdc.cancer.gov/) database, which included 48 tumor samples. We evaluated the correlation between RAB39B and immune-infiltrating cells using the ssGSEA algorithm in R (version 3.6.3) (Hanzelmann et al., 2013). The markers of immune cells drew on a report from Bindea et al. (2013). We also analyzed the expression level correlation between RAB39B and m6A-related genes in DLBCL samples and the differences in expression of m6A-related genes between the high and low RAB39B expression groups. M6A-related genes include YTHDC1, YTHDC2, IGF2BP1, IGF2BP2, IGF2BP3, YTHDF1, YTHDF2, YTHDF3, HNRNPA2B1, HNRNPC, RBMX, ZC3H13, METTL14, METTL3, RBM15, RBM15B, VIRMA, WTAP, FTO, and ALKBH5.

### LinkedOmics Analysis

The LinkedOmics database (http://www.linkedomics.org/login.php) is a web-based portal that can provide multi-omics data analysis for TCGA database and 10 Clinical Proteomics Tumor Analysis Consortium (CPTAC) cancer cohorts (Vasaikar et al., 2018). We searched for the differentially expressed genes related to RAB39B in DLBCL (*n* = 48) in the LinkFinder module. The Pearson correlation coefficient was used for statistical analysis of RAB39B co-expression. The correlation results were visualized by volcano plots and heat maps. RAB39B co-expression genes were annotated using Gene Ontology (GO) analysis and Kyoto Encyclopedia of Genes and Genomes (KEGG) pathway enrichment analysis via the LinkInterpreter module.

### STRING Analysis

STRING (https://cn.string-db.org/) is an online database that contains publicly available PPI data (Szklarczyk et al., 2019). In this study, we performed a PPI network analysis on RAB39B by STRING.

### ceRNA Network Construction

starBase (https://starbase.sysu.edu.cn/) is an open-source platform for studying RNA–RNA interactions from CLIP-seq, degradome-seq, and RNA–RNA interactome data (Li et al., 2014). We used starBase to predict the target miRNAs of RAB39B. Candidate miRNAs were derived from the overlap of PITA, miRanda, and TargetScan predictions. In addition, we analyzed the correlation between the expression of these candidates and RAB39B to screen for miRNAs that were more in line with ceRNA conditions.

miRNet2.0 (www.mirnet.ca/miRNet/home.xhtml) is a platform for miRNA-centric network visual analytics (Chang et al., 2020). We used starBase and miRNet2.0 to predict the target lncRNAs of hsa-miR-144-3p and hsa-miR-381-3p. Candidate lncRNAs were obtained from the overlaps of starBase and miRNet2.0. The correlation between the expression of lncRNA candidates and miRNAs was also analyzed to screen for lncRNAs that are more in line with ceRNA conditions. miRNA–mRNA and miRNA–lncRNA with negatively correlated expression levels were screened out to construct a key lncRNA–miRNA–mRNA (RAB39B) ceRNA network for DLBCL.

### Gene Set Cancer Analysis Analysis

Gene Set Cancer Analysis (GSCA, http://bioinfo.life.hust.edu.cn/GSCA/#/) integrates over 10,000 multi-dimensional genomic data from TCGA and over 750 small molecule drugs from GDSC and CTRP (Ji et al., 2016). Here, we investigated the correlation between RAB39B expression and drug response based on CTRP in the GSCA database.

### Clinical Tissues and Cell Culture

Lymph node clinical specimens were obtained from patients with newly diagnosed DLBCL and healthy donors at Third Xiangya Hospital. Fresh tissues were preserved in liquid nitrogen. All patients provided informed consent. Human DLBCL cell lines U2932 and OCI-LY7 were purchased from American Type Culture Collection (ATCC; Manassas, VA, United States). Cell culture was performed according to the recommended protocols. For small interfering RNA (siRNA) transfection, DLBCL cell lines were transfected with siRNA (Niu et al., 2020) (sense: 5′-UCA​UUC​UUC​AGA​AGA​GGU​UTT-3'; antisense: 5′-AAC​CUC​UUC​UGA​AGA​AUG​ATT-3′) using LipofectamineTM 3000 (Invitrogen, MA, United States).

### RNA Extraction and qPCR

Total RNA was extracted from clinical samples using standard TRIzol (Invitrogen, United States) RNA extraction protocol. RNA samples were quantified by NanoDrop ND-1000 (NanoDrop, United States). Reverse transcription was performed using PrimeScript RT Reagent Kit (Takara, China). Real-time qPCR was performed using Brilliant Ⅱ SYBR Green RT–qPCR kit. The 2^−ΔΔCt^ method was used for calculating relative levels of RAB39B. Primers used were as follows (Niu et al., 2020):

For RAB39B:

forward primer: 5′-CTG​GGA​TAC​AGC​GGG​TCA​AG-3′;
reverse primer: 5′-GAA​GGA​CCT​GCG​GTT​GGT​AA-3′.

For GAPDH:

forward primer: 5′- GGG​AAA​CTG​TGG​CGT​GAT-3′;
reverse primer: 5′- GAG​TGG​GTG​TCG​CTG​TTG​A-3′.

### Cell Proliferation Assay

For cell proliferation assay, cells were incubated in 96-well plates for the required time. Cell count kit-8 reagent (Sigma-Aldrich, St Louis, Missouri; 10 µL) was added to each well, followed by incubation for 4 h. The absorbance of the solution was measured at 450 nm using a microplate imaging system. For chemotherapeutic agent testing, doxorubicin (Sigma, 0.1 μM) or vincristine (MCE, 0.05 μM) was added 48 h after siRNA transfection. The cells were incubated for 72 h before monitoring the live cell rate with CCK-8.

### Flow Cytometry

Cells were washed with cold PBS and resuspended at a concentration of 1 × 10^6^ cells/ml in PBS containing 10% FBS and 1% sodium azide. For apoptosis analysis, apoptotic cells were detected using the Annexin V/PI Kit (KeyGEN BioTECH, China). Cells with the indicated transfection were collected and stained with Annexin V/PI in the dark for 15 min and subjected to flow cytometry. The analysis was performed with FACSVerse (BD Biosciences) and FlowJo software (Tree Star, United States).

### Statistical Analysis

All statistical analyses were performed in R (v3.6.3). Shapiro–Wilk normality tests were used to assess data normality. For expression data that obeyed normal distribution, statistical significance was assessed using an independent sample *t*-test. Otherwise, data were evaluated by the Wilcoxon rank sum test. In correlation analysis, the Pearson correlation coefficient was used to evaluate the correlation between normally distributed samples; otherwise, the Spearman correlation coefficient was used. The receiver operating characteristic (ROC) curve was used to analyze the possibility of RAB39B as a diagnostic marker. The survival package (v3.2-10) was used in R (v3.6.3) for statistical analysis of survival data. A *p*-value (two-tailed) < 0.05 was considered statistically significant.

## Results

### mRNA Levels of RAB39B in DLBCL and Pan-Cancer

**FIGURE 1:**
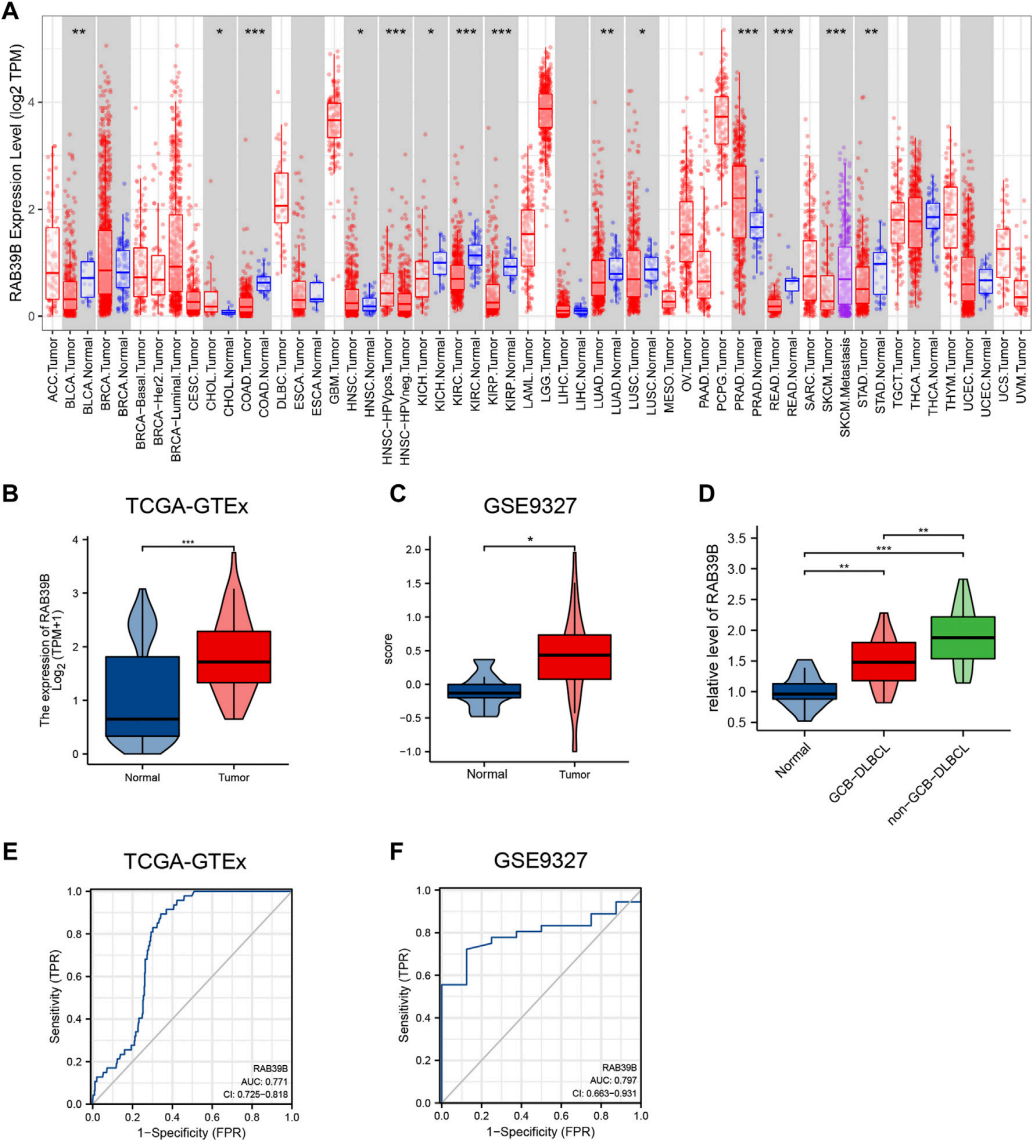
Relationship between RAB39B expression and DLBCL. **(A)** TIMER database showed dysregulation of RAB39B in various cancers. **(B)** Expression of RAB39B between DLBCL and normal controls in TCGA–GTEx. **(C)** GSE9327 dataset was used to assess the RAB39B level between DLBCL and normal tissues. **(D)** Clinical samples showed the elevated expression of RAB39B in GCB-DLBCL, and this trend was much more pronounced in non-GCB DLBCL patients. **(E,F)** ROC analysis illustrated that RAB39B expression accurately discriminated DLBCL tumor tissues from normal tissues with an AUC of 0.771 (95% CI: 0.725–0.818) from TCGA–GTEx data and an AUC of 0.797 (95% CI: 0.663–0.931) from the GSE9327 dataset. *p < 0.05, **p < 0.01, and ***p < 0.001.

The TIMER database was used to analyze the mRNA levels of RAB39B in tumors and adjacent normal tissues in pan-cancer. CHOL (cholangiocarcinoma), HNSC (head and neck squamous cell carcinoma), and PRAD (prostate adenocarcinoma) had significantly higher expression of RAB39B than adjacent normal tissues. In addition, HPV-positive HNSC had higher RAB39B than the HPV-negative subgroup. In SKCM (skin cutaneous melanoma), the RAB39B level was higher in metastases than in primaries. However, BLCA (bladder urothelial carcinoma), COAD (colon adenocarcinoma), KICH (kidney chromophobe), KIRC (kidney renal clear cell carcinoma), KIRP (kidney renal papillary cell carcinoma), LUAD (lung adenocarcinoma), LUSC (lung squamous cell carcinoma), READ (rectum adenocarcinoma), and STAD (stomach adenocarcinoma) had lower RAB39B expression than adjacent normal tissues (Figure 1A).

Since an adjacent normal control group of DLBCL is missing in TIMER, we analyzed TCGA-GTEx RAB39B expression data from the UCSC Xena database. The RAB39B mRNA level in DLBCL was significantly higher than that in normal tissues (Figure 1B). The clinical information is detailed in Supplementary Table S1. RAB39B mRNA levels were not associated with clinical stage, gender, extranodal involvement, and IDH level. The GSE9327 dataset from the GEO database also showed a significant increase of RAB39B in DLBCL tumor tissues compared with normal control (Figure 1C). For further validation, we collected and tested the level of RAB39B in 17 healthy donors with reactive lymph nodes and 33 DLBCL patients (including 15 GCB subtypes and 18 non-GCB subtypes). We noted the elevated expression of RAB39B in GCB-DLBCL, and this trend was much more pronounced in non-GCB DLBCL patients (Figure 1D). ROC curve analysis was used to evaluate the diagnostic effectiveness of the RAB39B mRNA expression level, which estimated the AUC (area under the curve) at 0.771 (95% CI: 0.725–0.818, Figure 1E) in the TCGA–GTEx data and 0.797 (95% CI: 0.663–0.931, Figure 1F) in the GSE9327 dataset.

### RAB39B PPI Network and Co-Expression Analysis in DLBCL

**FIGURE 2:**
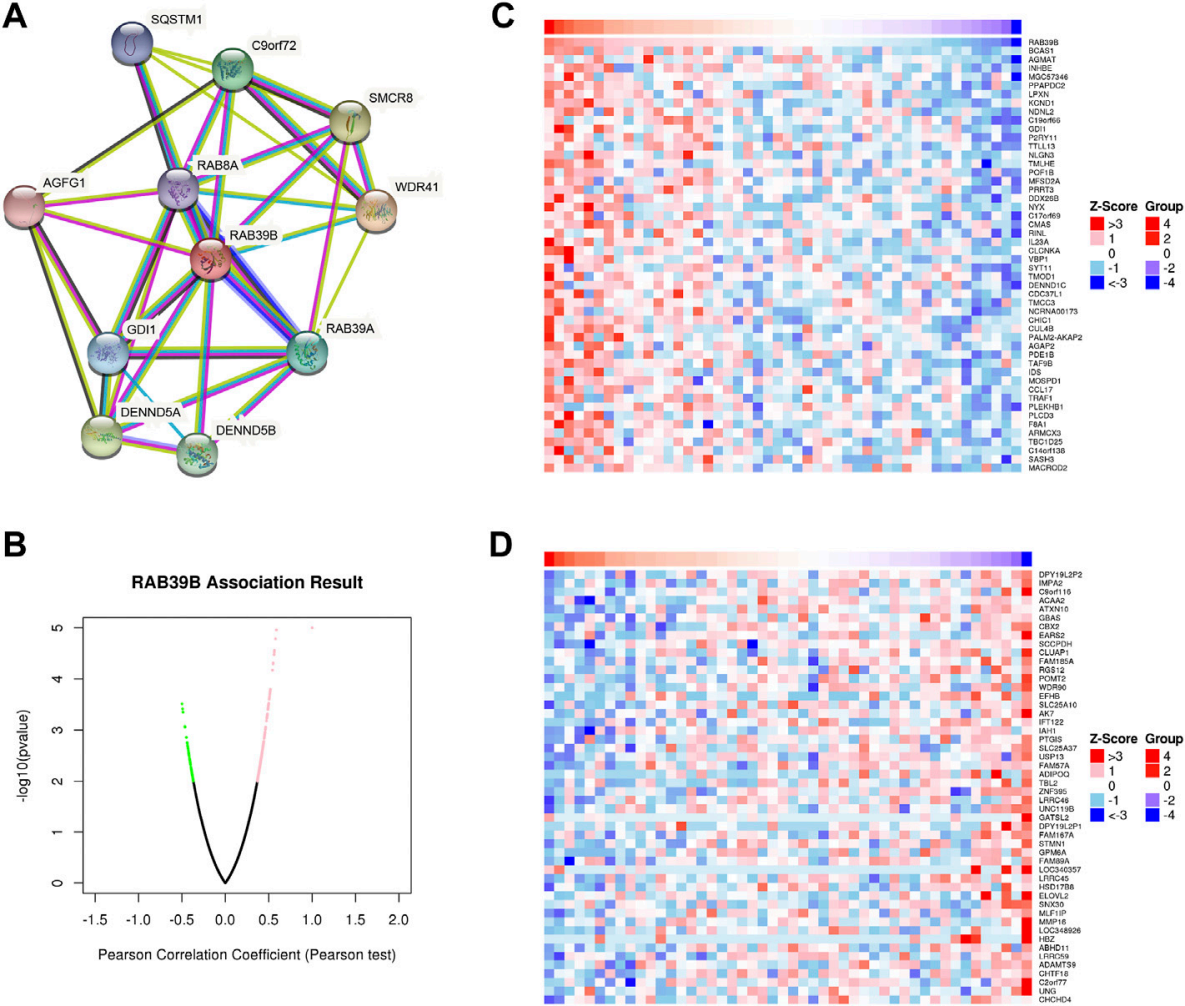
RAB39B PPI network and co-expression genes in DLBCL. **(A)** PPI network of RAB39B. **(B)** Genes highly correlated with RAB39B identified in DLBCL by the Pearson test in the LinkedOmics database. **(C)** Top 50 genes positively related to RAB39B in DLBCL. **(D)** Top 50 genes negatively correlated with RAB39B in DLBCL.

We used the STRING database to study the PPI network of RAB39B. The analysis showed that RAB39B was associated with WD repeat-containing protein 41 (WDR41), Smith–Magenis syndrome chromosome region, candidate 8 (SMCR8), DENN domain-containing protein 5A (DENND5A), C9orf72, DENN domain-containing protein 5B (DENND5B), RAB39A, Rab GDP dissociation inhibitor alpha (GDI1), sequestosome-1 (SQSTM1), RAB8A, Arf-GAP domain, and FG repeat-containing protein 1 (AGFG1) (Figure 2A and Supplementary Table S2. Among these proteins, WDR41, SMCR8, C9orf72, SQSTM1, and RAB39A are related to autophagy, and RAB8A is a member of the Ras-oncogene family.

**FIGURE 3:**
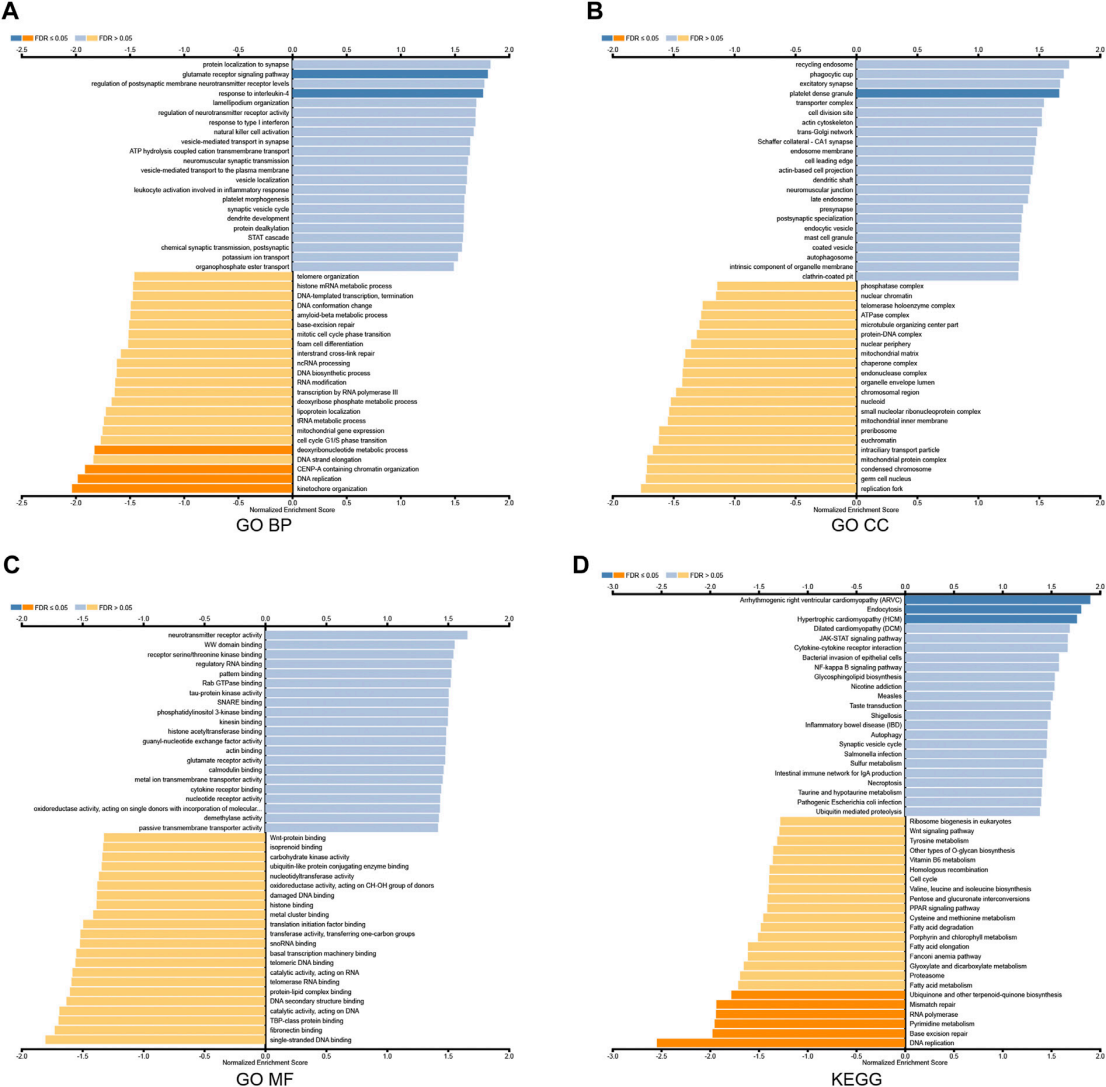
Enrichment analysis of RAB39B co-expression genes in DLBCL. **(A–C)** RAB39B co-expression genes were annotated by GO BP **(A)**, CC **(B)**, and MF **(C)** analysis. **(D)** KEGG pathway analysis of RAB39B co-expression genes.

To further explore the biological function, we used the LinkedOmics database to analyze RAB39B co-expression in DLBCL (Figure 2B). The heat map shows the top 50 significant genes that were positively correlated (Figure 2C) and negatively correlated with RAB39B (Figure 2D), respectively. The LinkInterpreter module was used to perform GO and KEGG enrichment analyses of RAB39B-related genes. The GO function annotation showed that RAB39B co-expression genes were mainly involved in DNA replication and protein synthesis (Figures 3A–C). KEGG pathway analysis indicated an enrichment in the cytokine–cytokine receptor interaction, JAK-STAT signaling pathway, NF-kappa B signaling pathway, and autophagy (Figure 3D). In summary, PPI analysis and enrichment analysis of the co-expression network suggested the potential role of RAB39B in DLBCL pathological processes. Further validation of the results will require more samples.

### RAB39B Was Associated With Immune Signatures in DLBCL

**FIGURE 4:**
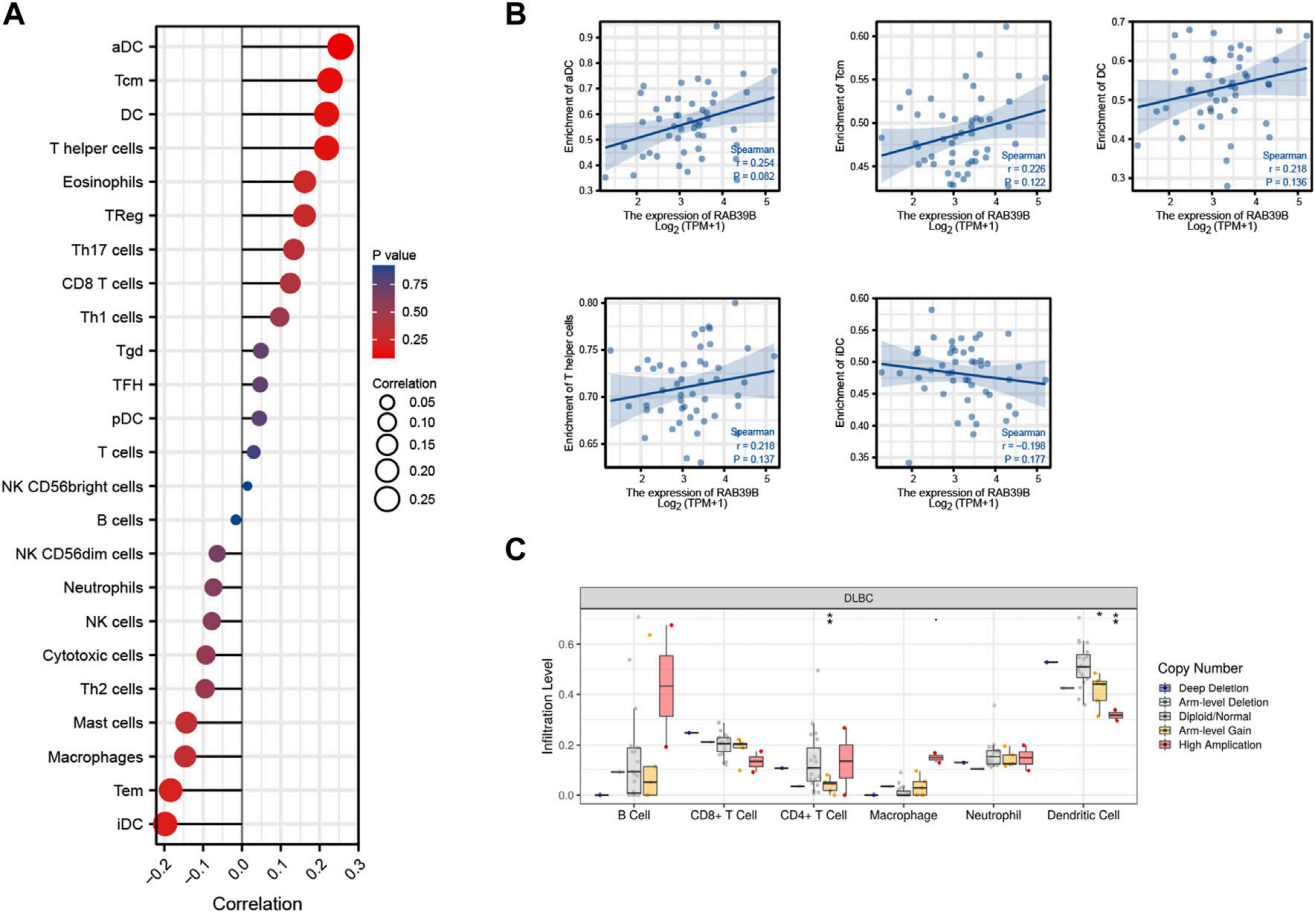
Relationship between RAB39B and immune signatures in DLBCL. **(A)** Relationship between RAB39B expression and 24 immune cell types. **(B)** Scatter plots of E2F2 expression with the immune infiltration level of aDC, Tcm, DC, T helper cells, and iDC. **(C)** RAB39B CNV affects the infiltrating levels of CD4^+^ T cell and DC in DLBCL.

We used TCGA expression data to evaluate the relationship between RAB39B expression and 24 different immune cell types in DLBCL. As shown in Figure 4A, RAB39B showed a close positive correlation with activated dendritic cells (aDCs), T central memory (Tcm), DC, and T helper cells and a negative correlation with immature dendritic cell (iDC). Although the difference does not seem to be statistically significant (Figure 4B), we thought it may have been impacted by the limited sample size. In addition, we also found that RAB39B CNV had a significant correlation with the infiltration level of CD4^+^ T cell and DC (Figure 4C). These results indicate that RAB39B plays an important role in the immune infiltration of DLBCL.

### RAB39B Was Associated With m6A RNA Methylation Regulators in DLBCL

**FIGURE 5:**
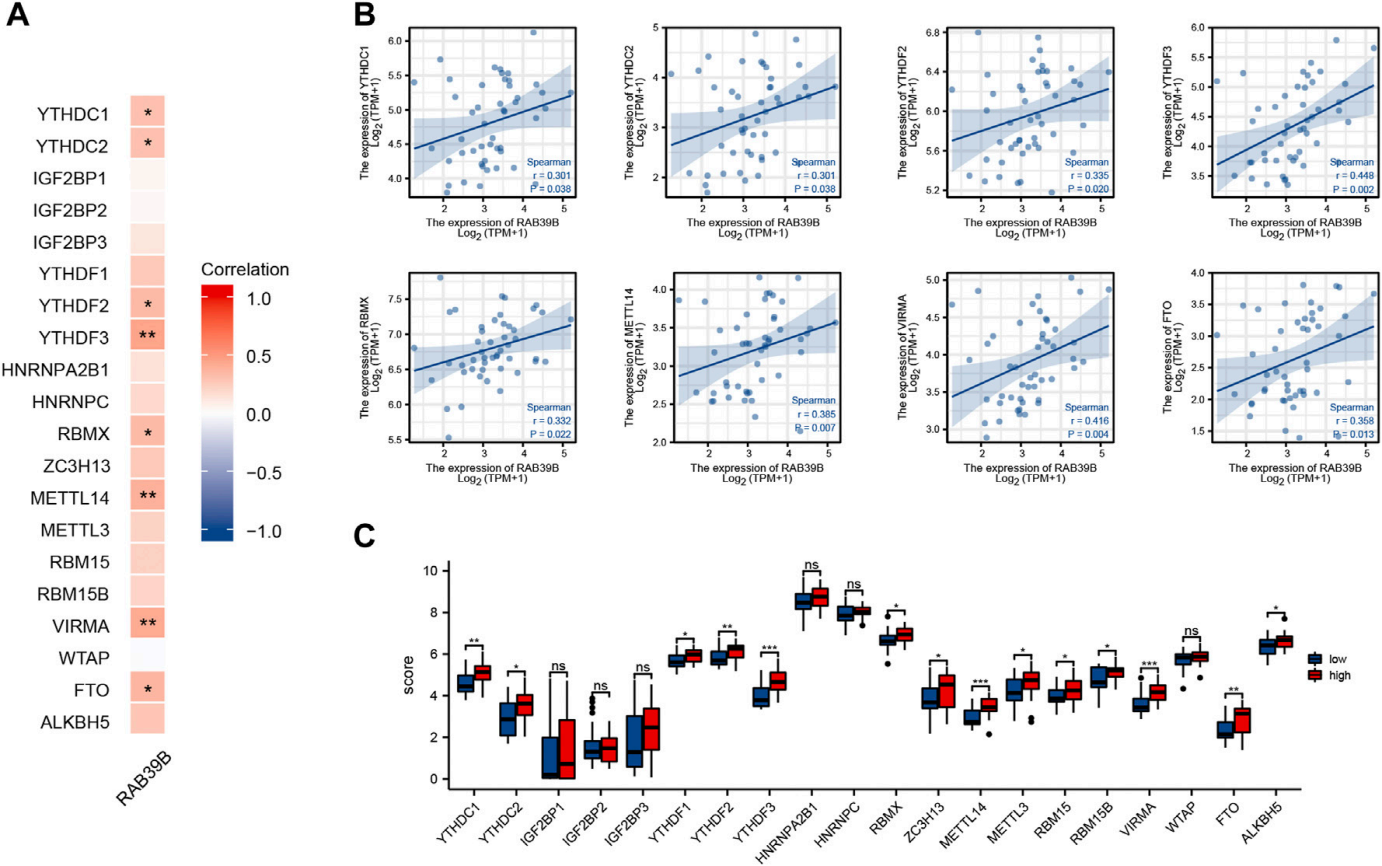
Correlation between RAB39B and m6A-related genes in DLBCL. **(A)** Correlation between expression of RAB39B and m6A-related genes in TCGA DLBCL cohort. **(B)** Scatter plots of the correlation between RAB39B and m6A-related genes. m6A-related genes include YTHDC1, YTHDC2, YTHDF2, YTHDF3, RBMX, METTL14, VIRMA, and FTO. **(C)** Variation among m6A-related genes in the high and low RAB39B expression groups in DLBCL.

We tried to analyze the correlation between the expression of RAB39B and 20 m6A-related genes by using TCGA DLBCL dataset (Figure 5A). As shown in Figure 5B, the RAB39B expression was significantly positively correlated with YTHDC1, YTHDC2, YTHDF2, YTHDF3, RBMX, METTL14, VIRMA, and FTO. In addition, 48 DLBCL samples were grouped by RAB39B expression, with 24 samples in the high-expression group and 24 samples in the low-expression group. We analyzed the differential expression of 20 m6A-related genes between different RAB39B expression groups in DLBCL. Compared with the low RAB39B expression group, the expression of YTHDC1, YTHDC2, YTHDF1, YTHDF2, YTHDF3, RBMX, ZC3H13, METTL14, METTL3, RBM15, RBM15B, VIRMA, FTO, and ALKBH5 were significantly upregulated in the high RAB39B expression group (Figure 5C).

### RAB39B-Related Key ceRNA Network Construction in DLBCL

**FIGURE 6:**
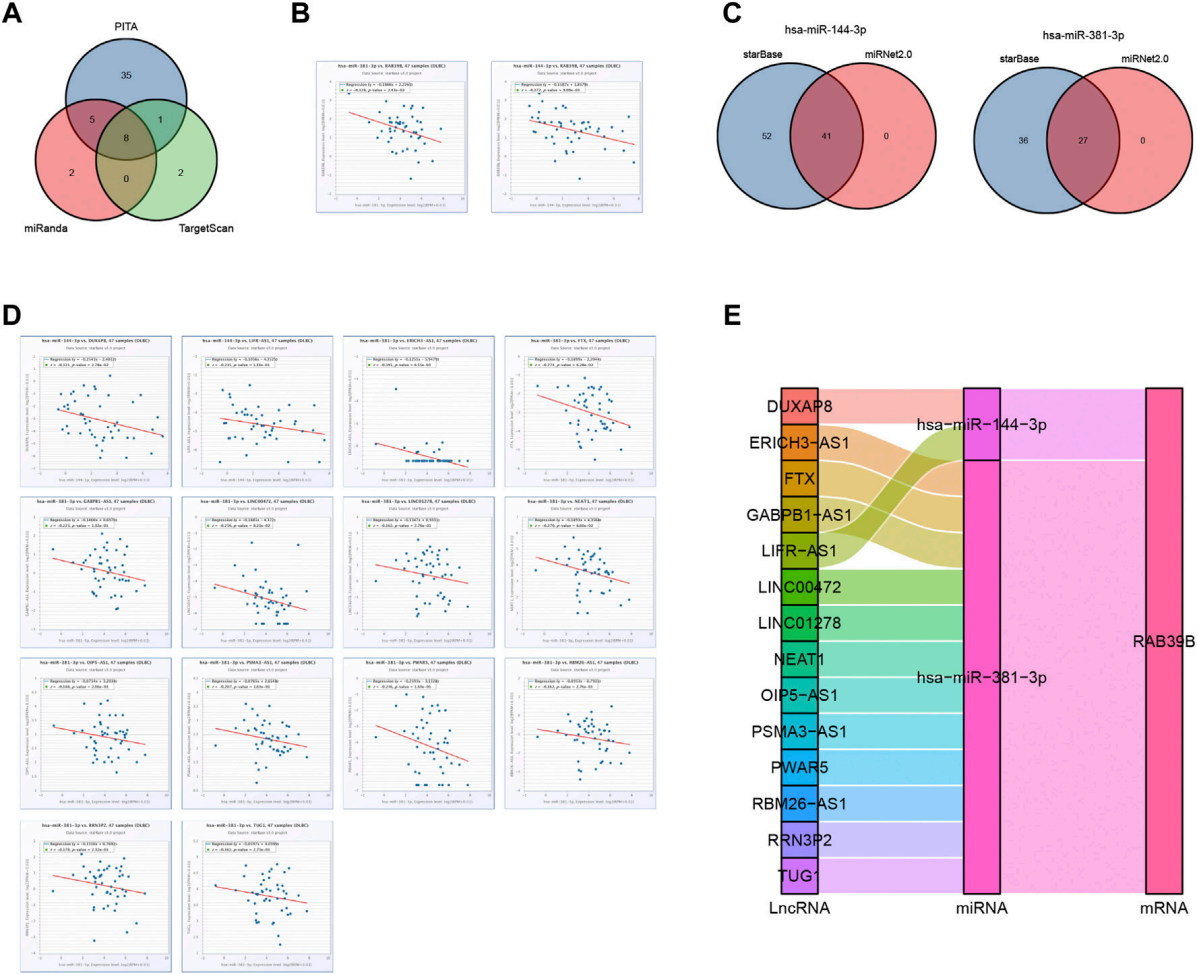
RAB39B-related key ceRNA network construction in DLBCL. **(A)** Venn diagram of predicted RAB39B targets in PITA, miRanda, and TargetScan. **(B)** Correlation between RAB39B and the target miRNAs in scatter plots. **(C)** Venn diagram of predicted target lncRNAs of hsa-miR-144-3p and hsa-miR-381-3p in starBase and miRNet. **(D)** Correlation between hsa-miR-144-3p or hsa-miR-381-3p and the target lncRNAs in scatter plots. **(E)** lncRNA–miRNA–mRNA (RAB39B) regulatory network in line with the ceRNA hypothesis was constructed in the Sankey diagram.

We used the starBase database to predict target miRNAs of RAB39B in PITA, miRanda, and TargetScan. We obtained 49, 15, and 11 RAB39B target miRNAs, respectively. In total, eight common miRNAs were predicted in these three databases (Figure 6A and Supplementary Table S3). Since miRNAs are generally considered to negatively regulate target genes, miRNAs negatively correlated with RAB39B were screened for ceRNA construction. We found that hsa-miR-144-3p and hsa-miR-381-3p were significantly negatively correlated with the expression of RAB39B in DLBCL (Figure 6B).

We used starBase and miRNet databases to further predict the lncRNAs that may bind to hsa-miR-144-3p and hsa-miR-381-3p and displayed the overlaps using the Venn diagram (Figure 6C; Supplementary Tables S4, S5). In the ceRNA network hypothesis, lncRNAs are generally negatively correlated with miRNAs. We performed correlation analyses and included lncRNAs having a correlation coefficient <0.15 with miRNAs. DUXAP8 and LIFR-AS1 were negatively correlated with hsa-miR-144-3p, and ERICH3-AS1, FTX, GABPB1-AS1, LINC00472, LINC01278, NEAT1, OIP5-AS1, PSMA3-AS1, PWAR5, RBM26-AS1, RRN3P2, and TUG1 were negatively correlated with hsa-miR-381-3p (Figure 6D). Finally, we constructed 14 pairs of ceRNA networks (Figure 6E).

### Effect of RAB39B Expression Level on Drug Sensitivity and Prognosis of DLBCL

**FIGURE 7:**
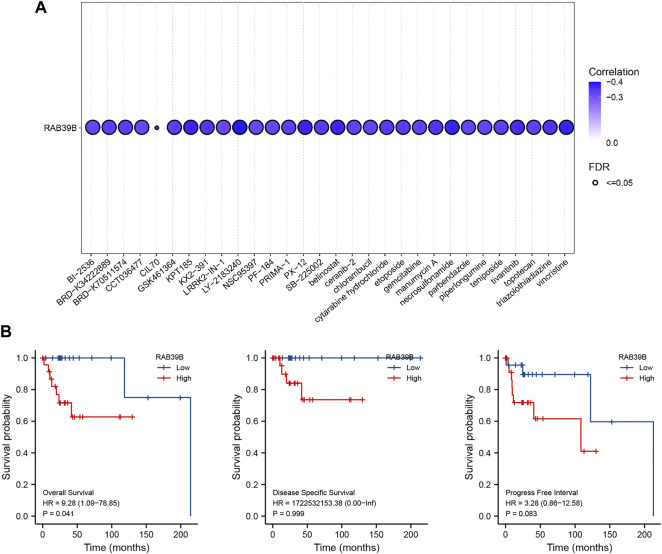
Clinical significance of RAB39B expression. **(A)** Correlation between RAB39B expression and the sensitivity of CTRP drugs (top 30) in pan-cancer. **(B)** Relationship between RAB39B expression and OS, DSS, and PFI using Kaplan–Meier survival analysis.

To further understand the clinical significance of RAB39B expression, we predicted the relationship between RAB39B level and drug sensitivity using the GSCA database. As shown in Figure 7A and Supplementary Table S6, RAB39B was negatively correlated with the sensitivity to several chemotherapeutic drugs commonly used in DLBCL, like dexamethasone (*r* = −0.20, FDR = 1.22 × 10^−6^), doxorubicin (*r* = −0.30, FDR = 2.36 × 10^−17^), etoposide (*r* = −0.33, FDR = 4.12 × 10^−21^), vincristine (*r* = −0.38, FDR = 2.64 × 10^−27^), and cytarabine (*r* = −0.34, FDR = 1.12 × 10^−21^).

We also assessed the impact of RAB39B expression on the survival of DLBCL. Kaplan–Meier analysis demonstrated that increased expression of RAB39B was significantly associated with poor overall survival (OS) [hazard ratio (HR) = 9.28, *p* = 0.041]. High expression of RAB39B was also associated with poor disease-specific survival (DSS) and progression-free interval (PFI), but the aforementioned differences were not statistically significant, which was probably because of a partial lack of survival data (Figure 7B).

### RAB39B Was Associated With DLBCL Cell Proliferation, Apoptosis, and Drug Sensitivity *In Vitro*

**FIGURE 8:**
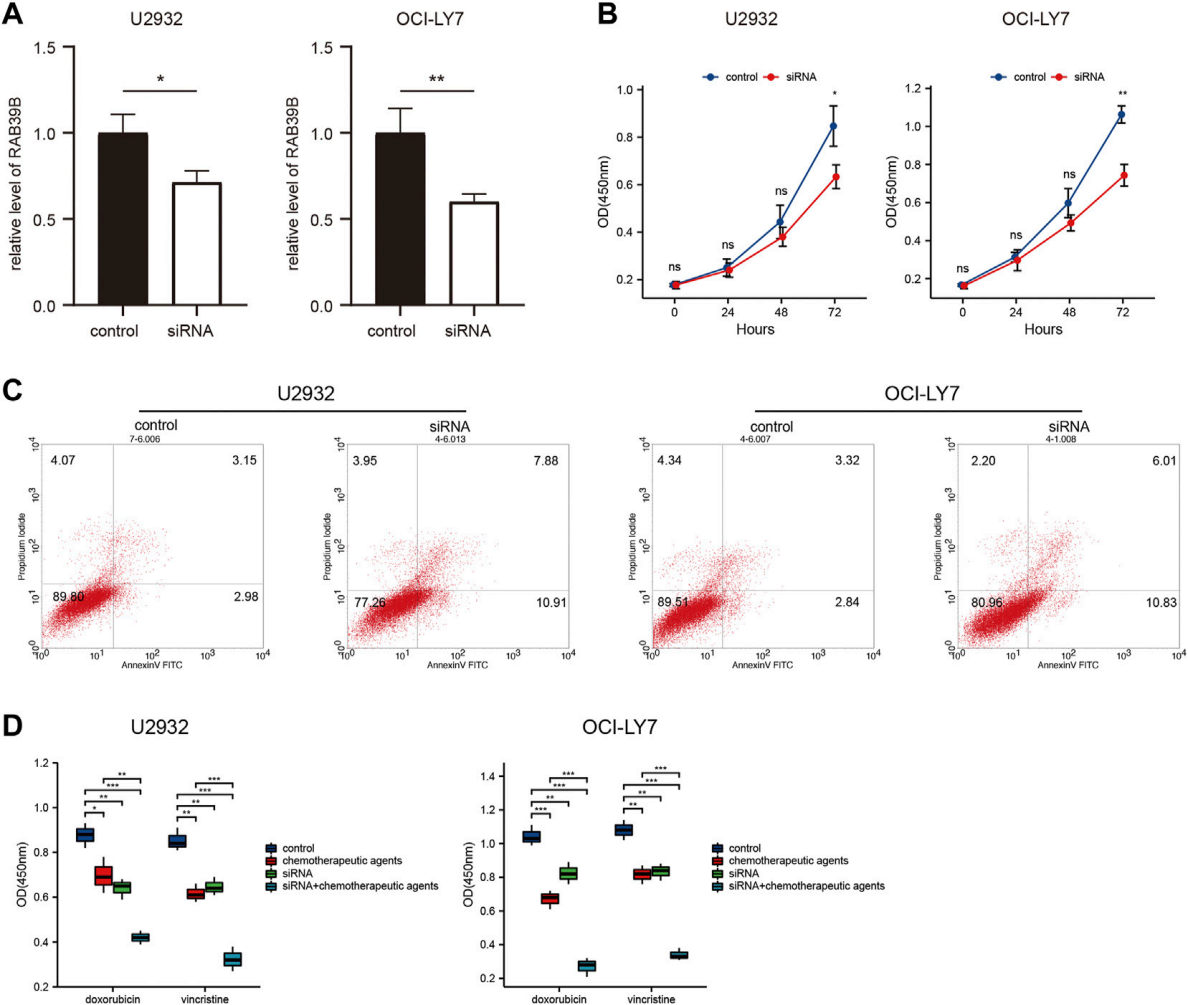
RAB39B was associated with DLBCL cell proliferation, apoptosis, and drug sensitivity *in vitro*. **(A)** RAB39B siRNA transfection efficiency in U2932 and OCI-LY7 was measured by qPCR. **(B)** CCK-8 proliferation assay showed that RAB39B inhibition resulted in decreased proliferation of U2932 and OCI-LY7 DLBCL cells. **(C)** Inhibition of RAB39B resulted in increased apoptosis in U2932 (18.79%) and OCI-LY7 (16.84%) DLBCL cells. **(D)** Inhibition of RAB39B significantly increased the sensitivity of DLBCL cells to doxorubicin and vincristine.

We validated the function of RAB39B *in vitro*. First, the expression of RAB39B in DLBCL cell lines U2932 and OCI-LY7 was inhibited by siRNA transfection (Figure 8A). CCK-8 proliferation assay showed that treatment with RAB39B siRNA resulted in decreased proliferation of DLBCL cells (Figure 8B). Inhibition of RAB39B also resulted in increased apoptosis in DLBCL cells (Figure 8C). In addition, inhibition of RAB39B increased the sensitivity of DLBCL cells to doxorubicin and vincristine (Figure 8D).

## Discussion

Rab proteins represent the largest family of the RAS superfamily of small GTPases with 66 members identified in the human genome. As major regulators of vesicular transport, Rab and Rab-associated factors have emerged as important regulators of cell growth, differentiation, survival, and programmed cell death or apoptosis (Li and Marlin, 2015). Rab dysregulation affects the regulation of multiple signaling pathways by disrupting membrane trafficking and therefore plays a driving role in diseases such as cancer, neurological disorders, and several other genetic disorders (Menasche et al., 2000; Wasmeier et al., 2006; Giannandrea et al., 2010; Li, 2011). Multiple abnormal Rab genes have been identified as oncogenic drivers in a wide range of cancers. For example, Rab1a is upregulated in colorectal cancer, and Rab3d is overexpressed in a series of tumors including breast and lung cancer. These abnormal Rab signals are considered to be closely associated with aggressive tumor phenotypes (Thomas et al., 2014; Yang et al., 2015). As mentioned previously, RAB39B is upregulated in cancer (Biermann et al., 2007; Kou et al., 2015). In this study, we analyzed the expression level of RAB39B using TCGA–GTEX, GEO, and TIMER data. To the best of our knowledge, we are the first to report on the expression and potential diagnostic value of RAB39B in DLBCL.

A subset of Rab proteins, as well as their guanine nucleotide exchange factors or GTPase-activating proteins, are involved in autophagy regulation. For example, Rab2, Rab5, Rab6, and Rab33B have been demonstrated to participate in different steps and settings of autophagy (Ayala et al., 2018; Ding et al., 2019; Morgan et al., 2019; Zhou et al., 2019). In RAB39B-knockout mice, RAB39B deficiency compromised autophagic flux at the basal level (Niu et al., 2020). On the other hand, deregulated autophagy has been linked to proliferation, apoptosis, and drug sensitivity found in DLBCL (Li L. J. et al., 2017; Xu et al., 2021). To date, the function of RAB39B in DLBCL remains unknown. Similarly, we found that RAB39B may be involved in the regulation of DLBCL autophagy through PPI and co-expression gene enrichment analysis. We also observed the enrichment of several cancer-related pathways, like cytokine–cytokine receptor interaction, JAK-Stat signaling pathway, and NF-Kappa B signaling pathway, using the KEGG pathway enrichment analysis of RAB39B co-expressed genes. These suggest the potential role of RAB39B in the initiation and development of DLBCL.

As a major component of the tumor microenvironment, immune infiltrates have been proven to contribute to tumor progression and immunotherapy responses. Tumor-infiltrating immune cells, especially T cells, serve as the cellular underpinnings of antitumor immunity. Studies also suggest the importance of other immune cells, including myeloid cells, B cells, and NK cells in cancer immunotherapies (Zhang and Zhang, 2020). The immune infiltrates in the microenvironment have been utilized to determine the prognosis of many solid cancers (Tobin et al., 2019; Gao et al., 2020). Therefore, a better understanding of infiltrating immune cells in the tumor microenvironment of DLBCL is essential for deciphering the mechanisms of immunotherapies, defining predictive biomarkers, and identifying novel therapeutic targets. In nodal DLBCLs, memory T cells, CD4^+^ T cells, and DC densities indicate a good prognostic value, whereas the influence of regulatory T cells (Tregs) is less clear. Immune infiltrate data for primary central nervous system DLBCL are very sparse. CD8^+^ cytotoxic T cells seem to provide a possible immune escape mechanism and are associated with poor prognosis in all DLBCLs. From previous studies, it is known that tumor-associated macrophages are not associated with a significant prognostic value (Galand et al., 2012). Immune infiltrate analysis showed that RAB39B was negatively correlated with iDC, Tem (T effector memory), and CD8 T-cell infiltration but positively correlated with Tcm, aDC, and DC in this study. CD4^+^ T cell and DC were also negatively correlated with CNV of RAB39B. These suggest that abnormality of RAB39B may lead to a poor prognosis of DLBCL by affecting immune infiltration. Further research is needed to understand alternative immune infiltration patterns.

As the most abundant eukaryotic mRNA modification, m6A is known to play a vital role in tumor initiation and progression by regulating target genes. The tumorigenic process in DLBCL is governed by both genetic and epigenetic aberrations. Previous studies have shown that m6A modifications are closely related to the occurrence and development of DLBCL. For example, Song et al. (2022) found that ALKBH5-mediated N^6^-methyladenosine modification of TRERNA1 promotes DLBCL proliferation. Cheng et al. (2020) revealed that the m6A methyltransferase METTL3 promotes DLBCL progression by regulating m6A in PEDF. piRNA-30473, a PIWI-interacting RNA, has been indicated to exert its oncogenic role in DLBCL by upregulating WTAP, an m6A mRNA methylase (Han et al., 2021). The cellular machinery that regulates m6A includes proteins acting as writers, erasers, and readers of m6A. We analyzed the relationship between RAB39B expression and common m6A readers (YTHDC1, YTHDC2, IGF2BP1, IGF2BP2, IGF2BP3, YTHDF1, YTHDF2, YTHDF3, HNRNPA2B1, HNRNPC, and RBMX), writers (ZC3H13, METTL14, METTL3, RBM15, RBM15B, VIRMA, and WTAP), and erasers (FTO and ALKBH5) in DLBCL. The RAB39B expression was significantly correlated with YTHDC1, YTHDC2, YTHDF2, YTHDF3, RBMX, METTL14, VIRMA, and FTO. In addition, the level of YTHDC1, YTHDC2, YTHDF1, YTHDF2, YTHDF3, RBMX, ZC3H13, METTL14, METTL3, RBM15, RBM15B, VIRMA, FTO, and ALKBH5 increased in the high RAB39B expression group. These results suggest that the *RAB39B* gene may be modified by m6A in DLBCL.

The theory behind the regulatory ceRNA network is based on the competitive binding of lncRNA or cirRNA with miRNA to affect mRNA expression. The ceRNA regulatory mechanism is evident in DLBCL. For example, Huang et al. (2021) found that LINC00857 contributes to the proliferation and lymphomagenesis of DLBCL by regulating the miR-370-3p/CBX3 axis. Miao et al. (2021) revealed that lncRNA GAS5 inhibits DLBCL cell proliferation by causing miR-18a-5p to upregulate RUNX1 expression. Zhu et al. (2019) illustrated that LncRNA SNHG16 promotes proliferation and inhibits apoptosis of DLBCL by targeting the miR-497-5p/PIM1 axis. In this study, we constructed an lncRNA–miRNA–mRNA (RAB39B) network based on miRNAs hsa-miR-144-3p and hsa-miR-381-3p, as well as lncRNAs DUXAP8, LIFR-AS1, ERICH3-AS1, FTX, GABPB1-AS1, LINC00472, LINC01278, NEAT1, OIP5-AS1, PSMA3-AS1, PWAR5, RBM26-AS1, RRN3P2, and TUG1. Among them, NEAT1 and TUG1 have been identified as tumor drivers in DLBCL (Cheng et al., 2019; Yuan et al., 2022). Here, we have uncovered the ceRNA network of RAB39B in DLBCL. Further experiments are required to validate this finding.

Finally, we explored the impact of the level of RAB39B expression on drug sensitivity and prognosis of DLBCL. Our results showed that increased RAB39B expression was associated with decreased sensitivity of commonly used chemotherapy drugs such as dexamethasone, doxorubicin, etoposide, vincristine, and cytarabine and poor OS in DLBCL. This trend verified the reliability of the previous functional analysis of RAB39B in DLBCL from a clinical perspective.

In summary, we are the first to analyze the relationship between RAB39B expression and tumor immune infiltrate, m6A modification, ceRNA network, drug sensitivity, and prognosis in DLBCL. RAB39B is abnormally elevated and associated with drug resistance and poor OS in DLBCL, which may be related to its involvement in immune infiltration, m6A modification, and regulation by multiple non-coding RNAs. Our study identifies RAB39B as an effective biomarker for the diagnosis and treatment of DLBCL.

## Data Availability

The original contributions presented in the study are included in the article/Supplementary Material; further inquiries can be directed to the corresponding author.
