# Supplementary material for: RAB39B as a Chemosensitivity-Related Biomarker for Diffuse Large B-Cell Lymphoma
Source: Front Pharmacol. 2022 Jul 15;13:931501. doi: 10.3389/fphar.2022.931501 (PMC9336119; doi:10.3389/fphar.2022.931501)
Supplement: Supplementary file 3 [file Table1.DOCX]

| Characteristic | Low expression of RAB39B | High expression of RAB39B | p |
| --- | --- | --- | --- |
| n | 24 | 24 |  |
| Clinical stage, n (%) |  |  | 1.000 |
| Stage I | 4 (9.5%) | 4 (9.5%) |  |
| Stage II | 8 (19%) | 9 (21.4%) |  |
| Stage III | 2 (4.8%) | 3 (7.1%) |  |
| Stage IV | 5 (11.9%) | 7 (16.7%) |  |
| Primary therapy outcome, n (%) |  |  | 0.702 |
| PD | 2 (4.3%) | 3 (6.5%) |  |
| SD | 1 (2.2%) | 2 (4.3%) |  |
| PR | 1 (2.2%) | 2 (4.3%) |  |
| CR | 19 (41.3%) | 16 (34.8%) |  |
| Gender, n (%) |  |  | 0.385 |
| Female | 11 (22.9%) | 15 (31.2%) |  |
| Male | 13 (27.1%) | 9 (18.8%) |  |
| Age, n (%) |  |  | 1.000 |
| <=60 | 13 (27.1%) | 14 (29.2%) |  |
| >60 | 11 (22.9%) | 10 (20.8%) |  |
| Extranodal involvement, n (%) |  |  | 0.787 |
| No | 11 (23.9%) | 14 (30.4%) |  |
| Yes | 11 (23.9%) | 10 (21.7%) |  |
| IDH level, n (%) |  |  | 0.721 |
| Normal | 5 (16.7%) | 8 (26.7%) |  |
| Abnormal | 8 (26.7%) | 9 (30%) |  |
| Age, mean ± SD | 58.67 ± 10.5 | 53.88 ± 16.59 | 0.239 |
